# Cysteine Oxidation in Proteins: Structure, Biophysics, and Simulation

**DOI:** 10.1021/acs.biochem.2c00349

**Published:** 2022-09-26

**Authors:** Diego Garrido Ruiz, Angelica Sandoval-Perez, Amith Vikram Rangarajan, Emma L. Gunderson, Matthew P. Jacobson

**Affiliations:** Department of Pharmaceutical Chemistry, University of California, San Francisco, California 94158, United States

## Abstract

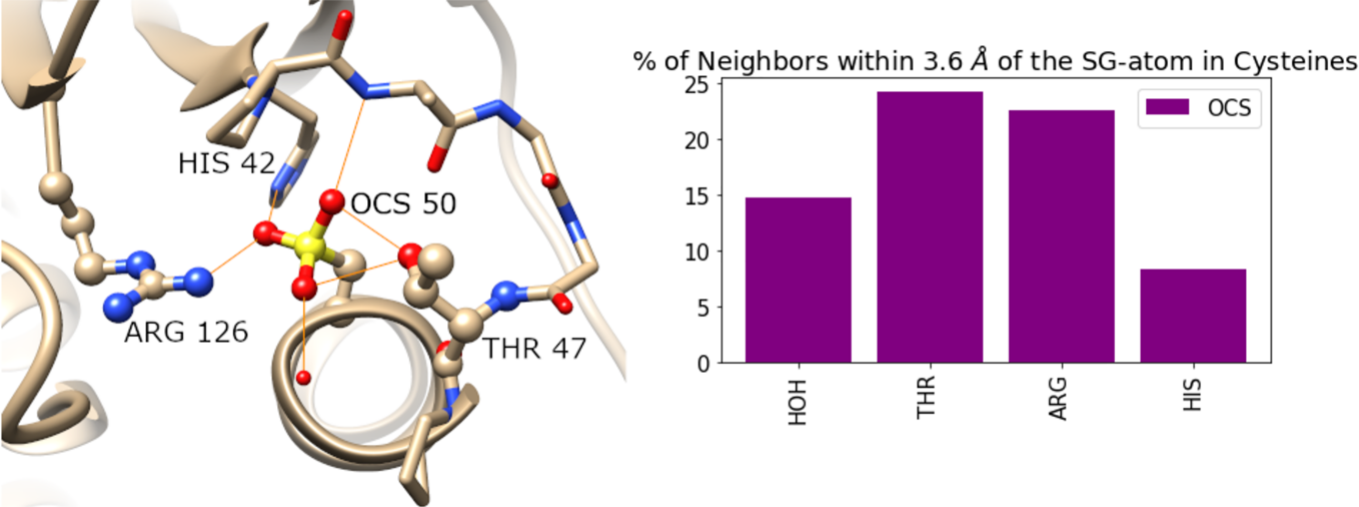

Cysteine side chains can exist in distinct oxidation states depending on the pH and redox potential of the environment, and cysteine oxidation plays important yet complex regulatory roles. Compared with the effects of post-translational modifications such as phosphorylation, the effects of oxidation of cysteine to sulfenic, sulfinic, and sulfonic acid on protein structure and function remain relatively poorly characterized. We present an analysis of the role of cysteine reactivity as a regulatory factor in proteins, emphasizing the interplay between electrostatics and redox potential as key determinants of the resulting oxidation state. A review of current computational approaches suggests underdeveloped areas of research for studying cysteine reactivity through molecular simulations.

Cysteine plays a uniquely important role in cellular responses to changes in the redox environment, such as those due to oxidative stress, with extensive links to pathological conditions such as neurodegeneration.^1^ One-electron oxidations of cysteine to radical species can occur, as well as two-electron oxidation to form disulfide bonds or acidic oxidized cysteine species as shown in Figure 1; only the latter will be considered here. We refer readers to a number of excellent review articles that examine thiol chemistry and proteomics methods for detecting cysteine oxidation in greater detail than we attempt to do here, as well as reviews of other important and related aspects of cysteine chemistry, such as disulfide bond formation and cysteine-reactive covalent ligands used as chemical biology probes or drugs.^2−7^

**Figure 1:**
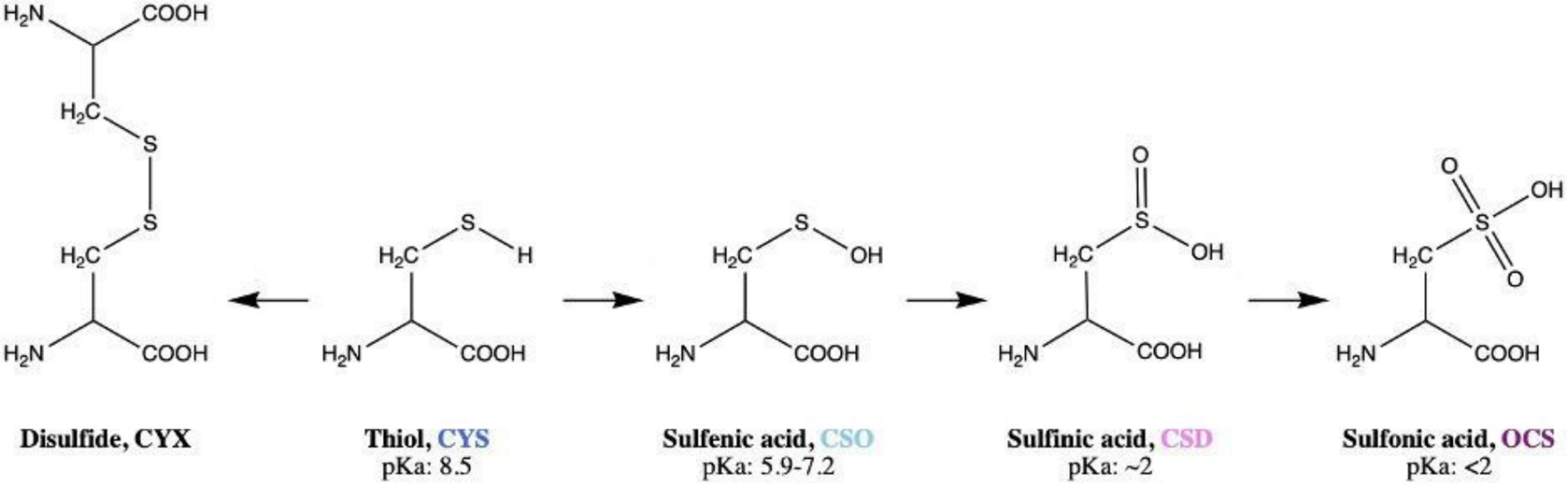
Primary oxidation states of cysteine and their p*K*_a_’s.^8,9^ Three-letter codes utilized by the Protein Data Bank are also provided.

Here we focus on aspects of cysteine oxidation that have received less attention, particularly insights from structural biology and other biophysical methods. In simple terms, our primary goals were to understand (1) what makes some cysteines more susceptible to oxidation than others, (2) trends and recurring motifs observed for the hydrogen bonding interactions of oxidized cysteines with other amino acids, and (3) the structural and dynamical consequences of cysteine oxidation in proteins, i.e., how these site-specific perturbations to the chemical structure modify the energy landscape, in ways that can ultimately impact function. In contrast to other post-translational modifications like phosphorylation,^10−14^ our understanding of oxidized cysteines, other than perhaps those involved in disulfide bonds, in the protein sequence–structure–function paradigm remains relatively rudimentary. While we attempt to advance this understanding, multiple challenges to doing so remain.

Unlike many other common post-translational chemical modifications of proteins, cysteine oxidations do not require catalysis by enzymes; subsequent reversal of these modifications (i.e., reduction) does, however, require enzymatic catalysis, except for reduction of cysteine sulfenic acid.^15^ The spontaneous oxidation of cysteines in response to changes in the redox state of the environment thus bears some resemblance to the spontaneous protonation or deprotonation of amino acid side chains due to changes in pH, leading to chemically minor changes that nonetheless significantly change biophysical properties and can thus impact function.^16−20^ The Nernst equation formalizes this analogy, providing quantitative estimates of the ratio of oxidized and reduced states given standard state reduction potentials (*E*^0^, analogous to the p*K*_a_) and the redox potential (*E*′, analogous to the pH)
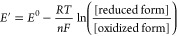
1as one can readily appreciate by comparison with the Henderson–Hasselbalch equation
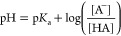
2

Units of the redox potential and standard state reduction potentials are conventionally reported in millivolts, emphasizing the origin of the Nernst equation in electrochemistry. A few biological redox processes, such as oxidative phosphorylation in mitochondria, permit an explicit analogy to electrochemical cells, but many other biological redox reactions, such as those carried out by most enzymes, limit the utility of this analogy. As an example, we refer readers to recent studies profiling the cysteine-mediated redox regulation of the actin cytoskeleton.^21,22^ We will not attempt to summarize the complex biochemical processes that together define the “redox state” of a cell and instead will simply state that it is not possible to describe, e.g., the cytoplasm of a cell using a single “redox potential”.^23,24^

Nonetheless, the Nernst equation provides a critical thermodynamic grounding, and vocabulary, for understanding cysteine oxidation. Glutathione is particularly relevant to the propensity of cysteines to become oxidized in intracellular proteins. As a highly abundant thiol-containing metabolite that can directly respond to redox conditions and participate in redox reactions, it functions as a redox buffer, and the ratio between oxidized (i.e., disulfide-linked glutathione dimers) and reduced glutathione defines a de facto redox potential for many redox reactions involving thiols. The standard state potential for glutathione disulfide reduction is approximately −240 mV at pH 7,^25,26^ but in the cytosol of mammalian cells in the absence of oxidative stress, concentrations of oxidized glutathione are kept low relative to those of reduced glutathione and thus the associated redox potential is even more negative, perhaps approaching −300 mV. In such a reducing environment, there is of course only a low probability for the formation of disulfides in proteins, as is well-known, or oxidation of cysteines to sulfenic acid or higher oxidation states. However, although relatively few direct measurements have been made, the reduction potentials for cysteines in proteins can be expected to vary significantly depending on their environment, just as the p*K*_a_’s of titratable amino acid side chains can vary by several units. For example, the redox potential for breaking the disulfide bond between Cys57 and Cys60 in the protein disulfide isomerase protein ERp57 was measured to be −167 mV, while that of Cys32–Cys35 in thioredoxin was −270 mV, both significantly shifted from the standard state reduction potential for glutathione disulfide of roughly −240 mV.^25^ One implication is that, while disulfide bonds are rarely found in the cytoplasm, they can form in some proteins, at least under conditions of oxidative stress. By the same token, the acidic oxidized forms of cysteine are likely generally rare in the cytoplasm, but hundreds of proteins with such modifications have been identified by mass spectroscopy experiments.^27−30^

Redox and pH are tightly intertwined with respect to the thermodynamics and kinetics of cysteine oxidation. Cysteine itself has a p*K*_a_ of ∼8.5, closer to cytoplasmic pH than those of any amino acid side chains except that of histidine.^31^ The formation of disulfide bonds generates two protons in addition to two electrons and thus depends on both pH and redox potential, as well as other factors.^32−34^ Rates of oxidation of the thiol side chain to sulfenic acid, as well as subsequent oxidations, are likewise pH-dependent (see ref (25) for a thorough study of mechanism), and sulfenic acid itself has a p*K*_a_ estimated to be roughly in the range of 6–7, such that both protonated and unprotonated species are likely to be present in the cytoplasm^35^ (the estimated p*K*_a_’s of sulfinic and sulfonic are <2, outside the physiological range^36−38^). The propensity to form the various oxidized states of cysteine can therefore vary substantially between different subcellular compartments with different pH and redox states, as well as dynamically as a function of cellular state, e.g., oxidative stress.

## Survey of Oxidized Cysteines in the Protein Data Bank

We analyzed all proteins in the Protein Data Bank (PDB) containing sulfenic, sulfinic, or sulfonic acids to investigate (1) the properties of the local environment of cysteines that may make them more or less susceptible to oxidation, (2) the preferred hydrogen bond interactions of oxidized cysteines, and (3) the structural consequences of cysteine oxidation.

A major limitation of this analysis is that nearly all of the relevant structures were obtained by X-ray crystallography, which itself can promote cysteine oxidation.^39^ This has two major implications. (a) The oxidized cysteines may not be biologically relevant; i.e., they can be viewed as artifacts of X-ray crystal structures. (b) The obtained structures may not represent any significant conformational changes that could occur in response to cysteine oxidation. In fact, among a relatively modest number of proteins for which structures have been obtained with the same cysteine in two or more different oxidation states, we observe relatively little conformational change (*vide infra*). The observation of cysteine sulfenic acid may present particular challenges because it can be rapidly oxidized or reduced non-enzymatically, depending on the redox and pH conditions. The transient nature of this modification may imply that its observation under nonphysiological conditions, such as in X-ray crystallography, could be biased relative to physiological conditions, e.g., toward cysteines with longer-lived oxidation states.

While we acknowledge these and other limitations, we believe that this analysis―to the best of our knowledge, the first large-scale attempt to characterize the local structural environment of oxidized cysteines―provides useful insights. Although the process by which cysteines are oxidized in X-ray crystal structures is distinct from cysteine oxidation in cells, we observe clear trends in the properties of the local environment that promote oxidation, as well as intriguing patterns of hydrogen bonding and other local interactions. Establishing the relevance of these observations to cysteine oxidation *in vivo* will require significant additional work, as we emphasize below.

The PDB was queried in March 2021 (March 10) for proteins containing oxidized cysteines, using the Biotite program^40^ and Prody package.^41^ The chemical component identifiers (CSO, CSD, or OCS) for the oxidized cysteines were used as search terms to find PDB entries with oxidized cysteines. These hits were filtered using cutoffs for structure resolution (<2.5 Å) and *R*_free_ values (<0.30); additional details are provided in Figure S1 and in a GitHub repository [Jacobson-lab-UCSF/Cysteine_oxidation: Cysteine oxidation in proteins: structure, biophysics, and simulation (github.com)]. The resulting 1124 structures represent <1% of the total structures in the PDB. In an attempt to consider the most biologically relevant oligomeric state, we constructed biological units for each of the PDB structures using the MakeMultimers.py script,^42^ which uses BIOMT transformation matrices provided in the REMARK 350 section of some PDB file headers, to construct multimer units of the protein. The first biological unit provided by the script was used for subsequent analysis, such as the discussion of hydrogen bond interactions below.

All PDB structures obtained from our searches were mapped to their respective UniProt accession codes to facilitate additional analyses. For example, at least one of the 15 UniProt keyword annotations (Table S1) for the cellular location of the protein was present for roughly half of the proteins in our data set (Table S2 and Figure S4). The small differences in these distributions should not be overinterpreted, due to multiple limitations of this analysis, including inconsistent reporting of the subcellular location. We note, however, that PDB structures containing cysteine sulfenic, sulfinic, and sulfonic acids are identified in proteins from essentially every subcellular compartment and/or localization.

## Do Proteins with Oxidized Cysteines in the PDB Also Have Oxidized Cysteines in Cells?

As a preliminary attempt to establish the potential biological relevance of cysteine oxidation observed in at least a subset of structures, we asked whether those proteins in our structural data set were also identified as containing oxidized cysteines in cells, using a variety of mass spectroscopy-based proteomics experiments. Specifically, a total of 4388 unique UniProt accession codes were extracted from the supplemental sections of several recent papers that identified proteins with cysteine sulfenic, sulfinic, or sulfonic acids.^29,43−47^ All of these proteins were from eukaryotic species, and 3436 (78%) were human in origin. We compared this set of UniProt ids against our database of oxidized cysteine structures obtained from the PDB. Of the 1124 crystal structures in our database, 349 are of human origin, and among these, nearly half (173, or 49.5%) were experimentally observed in at least one of the proteomics experiments (list provided in the GitHub repository). Only a minority of the proteomics data sets identifies specific oxidized cysteines, and thus, we have not attempted a more complete analysis at this time. There are, of course, limitations to the proteomics experiments, as well; e.g., low-abundance proteins are less likely to be detected. Proteomics studies may also be complicated by artifactual oxidation of cysteine and sulfenic acid and can detect alternative modifications such as perthiosulfenic acid and sulfenamide.^35,48−52^ Conversely, physiologically relevant, transient oxidation to sulfenic acid may in some cases not be detected.

## Local Environment of Cysteine Sulfenic, Sulfinic, and Sulfonic Acid Side Chains

**Figure 2:**
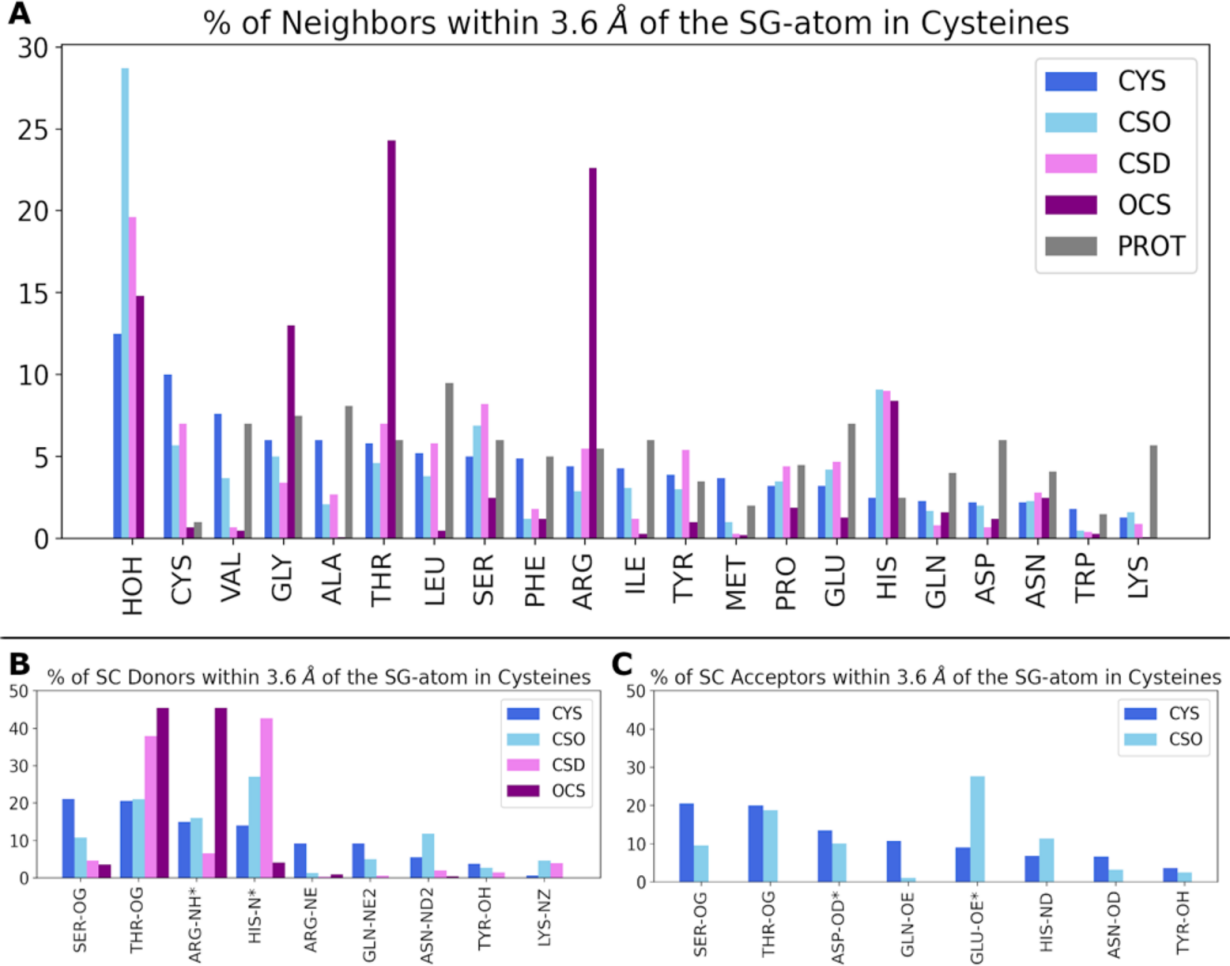
Local environment of different cysteine side chain oxidation states. (A) Neighboring amino acids (and crystallographic waters) within a cutoff of 3.6 Å around the sulfur atom (SG), compared to the average abundance of amino acids from proteins in the Protein Data Bank (“PROT”, gray).^53^ The amino acids are arranged along the *x*-axis in decreasing order for the CYS distribution; i.e., an unmodified CYS is most likely to be found near a crystallographic water or another CYS, and least likely to be found near Trp or Lys. (B) Side chain hydrogen bond donors within 3.6 Å of the sulfur. (C) Side chain hydrogen bond acceptors within 3.6 Å of the sulfur. Data for hydrogen bonds involving backbone amide groups are presented in Figure S3.

The 1124 structures in our data set contain a total of 1171 sulfenic acids (CSO), 469 sulfinic acids (CSD), and 382 sulfonic acids (OCS). In addition, these same structures also contain 7103 other cysteines that are not assigned as being oxidized or otherwise modified, i.e., presumed to be thiol or thiolate (CYS). Cysteines assigned as participating in a disulfide bond (CYX) were excluded from this analysis. We compared the local environments of the various states of cysteine by extracting all atoms within 3.6 Å of each sulfur atom, within the assumed biological assembly as discussed above. Figure 2 summarizes the probability of finding a given amino acid (or crystallographic water) in the proximity of the sulfur atom of a cysteine, normalized by the average number of neighbors. We also specifically identify hydrogen bond donors and acceptors in the immediate vicinity of the side chains in panels B and C of Figure 2.

The physicochemical properties of unmodified cysteine, including its hydrogen bond interactions, have been discussed extensively.^54−58^ In brief, while the cysteine side chain can act as a hydrogen bond donor (thiol) or acceptor (thiolate or thiol), and frequently does so with, e.g., backbone amide groups, the cysteine side chain is frequently found in hydrophobic environments. This propensity likely reflects both physicochemical properties and biological selection related to its reactivity. With respect to the latter, the low frequency of lysines (but not arginines) in the proximity of cysteine side chains, independent of oxidation state, is notable. A possible explanation is provided by recent work showing that Cys and Lys side chains can form redox-sensitive covalent linkages, which in turn can regulate enzymatic activity.^59^ Thus, the low prevalence of Lys around Cys may reflect evolutionary selection against forming such covalent linkages, which could be deleterious to the function of many proteins.

The sulfenic, sulfinic, and sulfonic acid side chains of oxidized cysteine are clearly more strongly polar than the unmodified (thiol) side chain, and unsurprisingly, these oxidized cysteine side chains are found much less frequently next to hydrophobic amino acids like Val, Ala, Leu, Phe, Ile, Met, and Trp. The highest oxidation state of cysteine, sulfonic acid (OCS), shows this trend most reliably, and the intermediate oxidation states (CSO and CSD) show this trend to a somewhat lesser extent.

Conversely, polar interactions, on average, increase for the oxidized cysteine side chains, but not uniformly. Histidine exhibits one of the most striking trends. The probability of finding a His around non-oxidized cysteines (CYS) is close to the average prevalence of His observed in proteins but increases nearly 3-fold around any of the oxidized states of cysteine (CSO, CSD, or OCS), as shown in Figure 2A. We hypothesize that this can be explained by histidine acting as a proton acceptor, leading to a decrease in the p*K*_a_ of the neighboring cysteines and making them more liable to oxidation.

Crystallographic waters are also more likely to be identified near CSO and CSD. We further examined the solvent accessibility of the various states of cysteine by computing the solvent accessible surface area (SASA) of each cysteine from the data set of 1124 proteins, using the tool *get_sasa_relative* from Pymol API version 2.4.2. The SASA values are normalized by the SASA for the fully solvent exposed amino acid, such that a relative SASA value of 0 represents a completely buried amino acid and 1 represents full solvent exposure.

**Figure 3:**
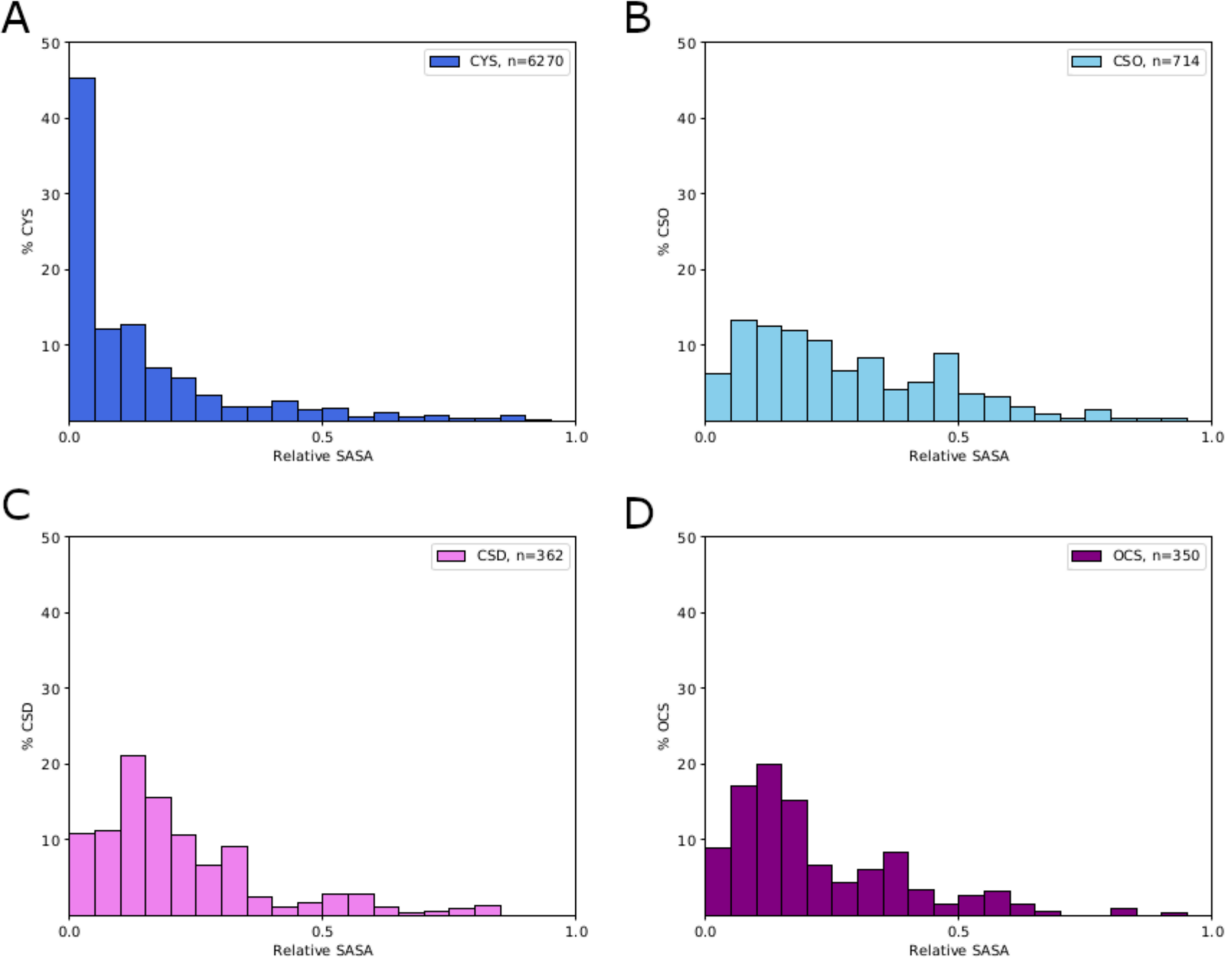
Solvent accessible surface area relative to the total surface area (relative SASA) of cysteines in various oxidation states.

**Figure 4:**
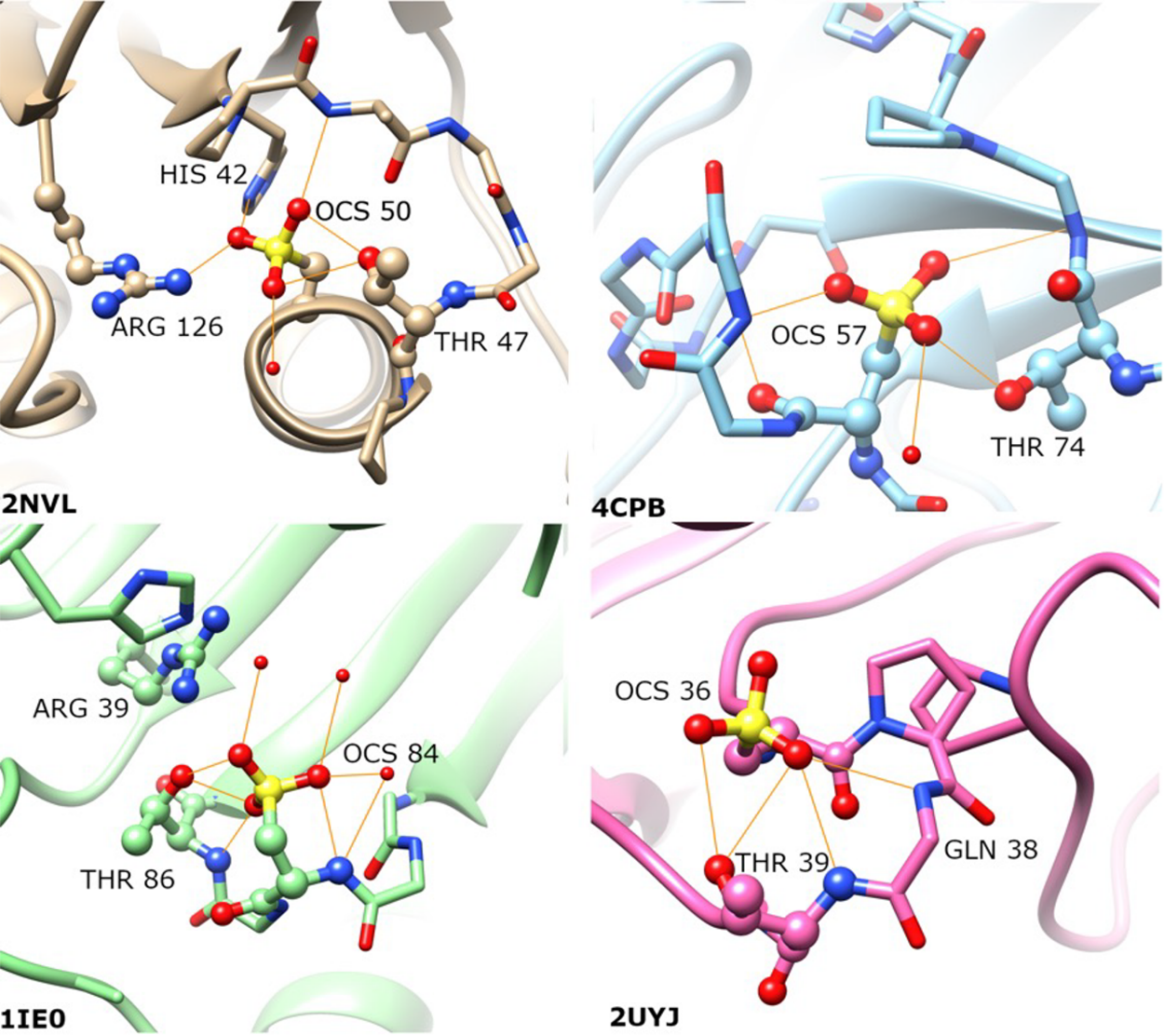
Representative examples illustrating the structural neighborhoods of sulfonic acids (OCS), highlighting hydrogen bond interactions (orange) with Thr side chains, as well as backbone amides and Arg side chains. Peroxidatic cysteine in the sulfonylated state is located at the N-terminus of an α helix (PDB entry 2NVL) (top left). In the remaining cases, the sulfonic acid is located on a loop: (top right) LecA (PDB entry 4CPB), (bottom left) LuxS (PDB entry 1IE0), and (bottom right) TdcF domain (PDB entry 2UYJ).

As expected on the basis of previous work, non-oxidized cysteines (CYS) are commonly found fully or partially buried in the protein (Figure 3A). All of the oxidized forms show much greater solvent exposure, on average, although even the highest oxidation state, sulfonic acid, tends to remain partly buried, due in part to its tendency to form multiple hydrogen bonds [*vide infra* (Figure 4)]. Overall, sulfenic acid (CSO) shows the greatest solvent exposure, on average, in agreement with the observation that crystallographic waters are most commonly found around CSO.

An unexpected, and to the best of our knowledge previously unreported, structural motif observed in this work is that some combination of Thr, Arg, and Gly is commonly found in the proximity of sulfonic acid (OCS). Specifically, we identified this structural motif in archaeal, bacterial, and eukaryotic peroxiredoxins [seven structures (2NVL, 2CV4, 5XBR, 1XIY, 4D73, 5OVQ, and 5IMV)], for which the role of cysteine oxidation in the antioxidant activity of the enzymes has been discussed in detail,^60^ as well as LuxS enzymes (PDB entries 1JVI, 1JQW, 1J98, and 1IE0), lectin domains (PDB entries 4CP9 and 4CPB), TdcF domains (PDB entries 2UYJ and 2UYK), and a nitrate reductase (PDB entry 3O5A). Representative examples are depicted in Figure 4, highlighting how the sulfonic acids act as hydrogen bond acceptors to the Thr and Arg side chains, and backbone amides from Gly.

It is of course not surprising that sulfonic acid would act as a hydrogen bond acceptor. Rather, the surprising aspects are the apparently strong preference for Thr side chains as a hydrogen bond partner but not Ser, the preference for Arg versus Lys (discussed above), and to some extent the extensive network of hydrogen bonds, with all three oxygens involved in at least one hydrogen bond each, in most cases. As a point of comparison, the post-translationally modified amino acid sulfo-tyrosine also commonly acts as a hydrogen bond acceptor for backbone amide groups but shows a strong apparent preference for hydrogen bonds with Lys rather than Arg.^61^ We emphasize, however, that these observations concerning sulfonic acid, as well as several of the other trends discussed above, should be considered preliminary at this point, because there are simply not enough distinct examples to reach statistically rigorous conclusions, in addition to other caveats discussed above.

Our analysis of PDB structures is complemented by previous work quantifying the rates of oxidation of various cysteines in specific purified proteins with H_2_O_2_, a physiologically relevant oxidant. For example, Weerapana et al.^27^ studied reactive cysteines in glutathione *S*-transferase GSTO1, acetyl-CoA acetyltransferase-1 (ACAT1), D15Wsu75e, and protein arginine methyltransferase PRMT1. Among these, ACAT1 has the most relevant structural information because three PDB structures (2IBU, 2IBW, and 2IBY) have been determined with one of the relevant cysteines being assigned as CSO (cysteine sulfenic acid). Consistent with the various caveats discussed above, the nucleophilic Cys126 was actually found to have a oxidation rate lower than those of Cys119, Cys196, and Cys413, which were assigned as being non-oxidized in the structures. The environment of Cys126 appears to exemplify the influence of a neighboring histidine, which we expect would shift its p*K*_a_ and favor the oxidation to CSO (Figure S5). While Cys119 is assigned as being non-oxidized in all PDB structures, its p*K*_a_ is also likely shifted by the nearby Arg105. Similarly, Cys413 is in the structural proximity of Cys126 and thus shares a similar physicochemical environment. We cannot account, however, for the apparently rapid *in vitro* oxidation of Cys196, which appears to occupy a highly hydrophobic environment not expected to favor oxidation. We speculate that conformational dynamics in solution could account for this discrepancy.

## Structural, Dynamical, and Functional Consequences of Cysteine Oxidation

The impact of post-translational phosphorylation on protein structure, dynamics, and function is, by now, well studied. Pairs of structures of the same protein, with and without site-specific phosphorylation, provided early insights into how the energy landscape of a protein could be perturbed by post-translational chemical modification,^62,63^ impacting catalytic activity and protein–protein interactions, for example. Other post-translational modifications, such as Lys acetylation, have also received considerable attention from the standpoint of protein structure and function. Cysteine oxidation, like phosphorylation or acetylation, significantly changes amino acid properties (as suggested in the preceding section) in a site-specific way, which can in principle drive changes in structure and function, but as emphasized in the introduction, there are substantial challenges to developing this understanding.

There is ample evidence from cellular biology that cysteine oxidation plays important roles in regulating pathways and individual proteins. For example, the mechanism of redox regulation in the epidermal growth factor (EGF) signaling pathway has been shown to be mediated by oxidation of a set of cysteine residues in a specific concerted manner, leading to EGF-dependent phosphorylation and growth factor signaling.^64^ Oxidation of catalytic cysteines of protein tyrosine phosphatases^65^ leads to their inactivation, while oxidation of cysteines in the kinase domain of EGFR increases its activity.^64,66,67^ Redox regulation of other kinases such as PKC,^68^ Src,^35,69^ Akt2,^70^ and Aurora A^71^ has been demonstrated, as well as several other phosphatases,^72,73^ transcription factors,^74,75^ ion channels,^76,77^ mitochondrial transporter proteins,^78,79^ and cytoskeletal proteins.^21,80−82^ However, in most of these cases, a detailed structural understanding of the mechanism of regulation by cysteine oxidation is lacking. One notable exception is a recent study that combined molecular dynamics simulations, biochemistry, and cell biology to develop a detailed mechanistic model for Src regulation by oxidation of cysteine to sulfenic acid.^35^

Perhaps the best studied human protein from the perspective of the structural and functional impact of cysteine oxidation is DJ-1,^83^ the protein product of the PARK7^84^ gene linked to autosomal recessive early onset Parkinson’s disease. DJ-1 has been shown to respond to oxidative stress with an increase in the level of acidic protein isoforms,^85^ mediated by the oxidation of one of three conserved redox-sensitive cysteines (Cys46, Cys53, and Cys106). An important role for oxidative stress is well established in the pathophysiology of Parkinson’s disease and other neurodegenerative disorders. DJ-1 is hypothesized to play a protective role in the cellular response to oxidative stress, and the disease-causing mutations in DJ-1 are believed to convey loss of function by mechanisms that remain poorly characterized.

**Figure 5:**
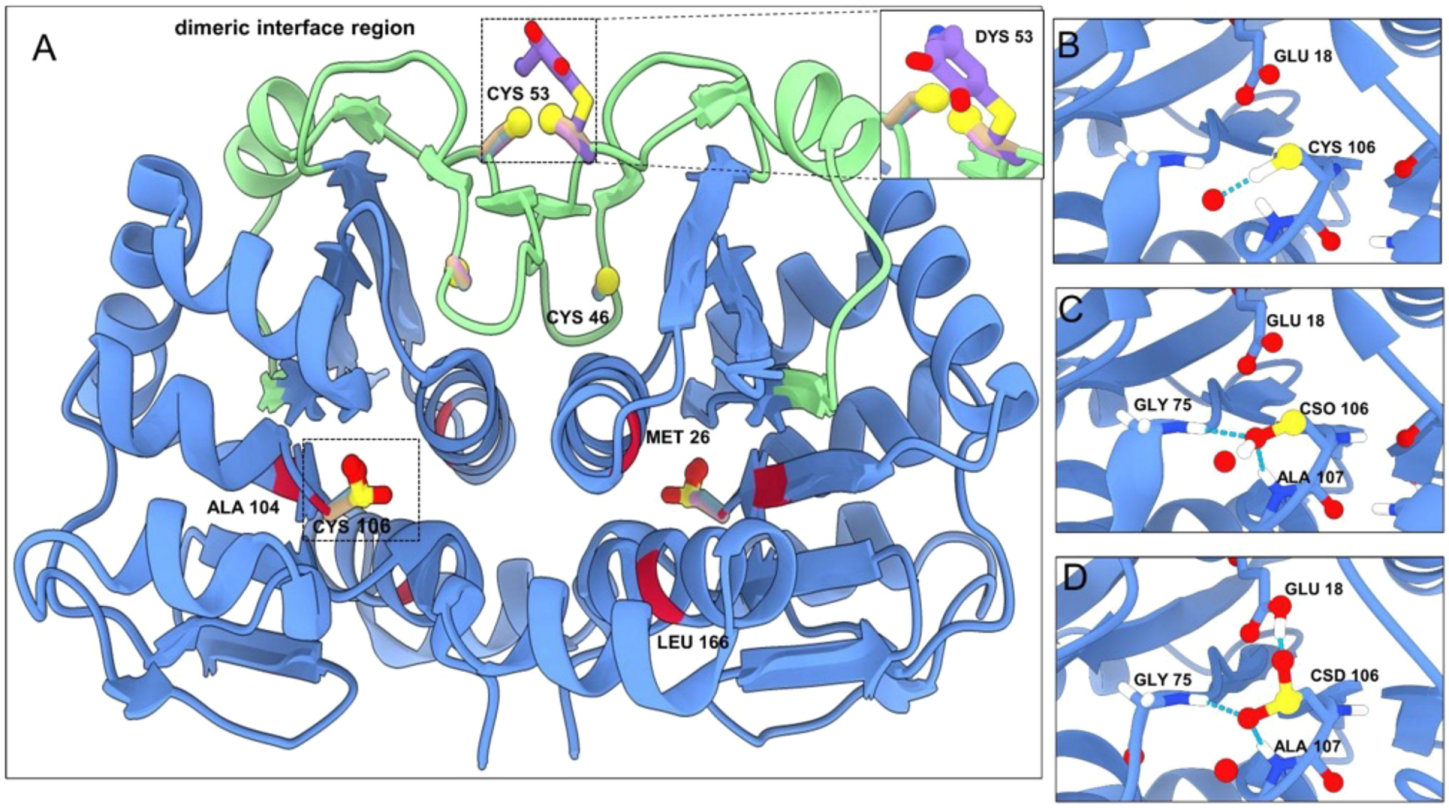
DJ-1 structures with cysteines in different oxidation states (PDB entries 1SOA, 4RKW, 4P34, and 2R1T), with the dimeric interface region (residues 38–70) colored green. The cysteines in the structure are shown in ball-and-stick representation labeled with their respective residue numbers. The regions enclosed in the dashed box containing Cys106 in different oxidation states (-SH, -SOH, and -SOOH) are enlarged in panels B–D. The positions associated with familial mutations are colored red on the cartoon representation with their residue numbers shown. Cys53 can undergo dopamine quinone conjugation (inset in the top right corner of panel A).

The precise molecular function of DJ-1, unfortunately, remains elusive, with various authors describing it, with varying degrees of plausibility, as a chaperone,^86^ an enzyme, or an antioxidant. Our own view is that DJ-1 can be described as a redox-sensitive signaling protein, regulating partners such as Nrf2^87^ and ASK1^88,89^ in a cysteine oxidation-dependent manner, analogous to calmodulin as a calcium-dependent signaling protein. What has been clearly established is the central role of Cys106 oxidation in its cellular function. From a structural perspective, Cys106 is located on a sharp turn between a β strand and a helix (“nucleophilic elbow”^90^) likely contributing to the reactivity of this cysteine.^91^ Cys106 is highly conserved among homologues of DJ-1, and the C106A substitution greatly reduces the extent of formation of oxidized isoforms.^85^ The sulfinic acid form of Cys106 (CSD) is considered to be the most active with respect to the role of DJ-1 in responding to oxidative stress, while both the reduced form (thiol) and sulfonic acid (OCS) forms are inactive. Crystal structures have also been determined with Cys106 in three different oxidation states (thiol, sulfenic acid, and sulfinic acid) (see Figure 5); we were unable to identify any other protein for which structures have been determined with more than two different states of Cys. Minimal conformational changes are observed to accompany oxidation of Cys106, but the role of Glu18 is noteworthy, with its protonation state apparently changing depending on the oxidation state of Cys106.^87,92,93^ Mutation of Glu18 also impacts the oxidation of Cys106, and more speculatively, some disease-associated missense mutations are also found close to Cys106 (e.g., M26I and A104T) and could exert their effects, in part, by modulating the oxidation of Cys106.^94,95^

Both Cys46 and Cys53 appear also to contribute to DJ-1’s ability to sense and respond to oxidative stress, although their roles are less clear.^96,97^ Cys53 is intriguing because it is located at the dimer interface of DJ-1 and appears to be responsible for covalently linked DJ-1 dimers,^98^ which have been identified in neuropathology studies of patients with neurodegenerative diseases,^99^ possibly through disulfide formation across the dimer interface.^100^ Moreover, a recent crystal structure demonstrated that Cys53 can form covalent adducts with dopamine quinone,^101^ hinting at a possible role of DJ-1 in responding to reactive dopamine species created by oxidative stress. A recent proteome-wide study profiled the reactivity of dopamine quinones and potential implications in disease, suggesting that this mode of regulation may be more widespread.^102^ While the roles of Cys53, as well as the potential roles of methionine oxidation and other redox modifications to DJ-1,^99^ are outside the scope of this review, we briefly mention them here to emphasize the complexity and richness of oxidative chemical modifications of DJ-1, and potentially other proteins as well, requiring further study. We recommend the excellent review by Wilson for a more in-depth discussion of DJ-1 biology.^103^

Although there are currently few cases for which crystal structures have been determined with cysteine oxidations that are well characterized to be important *in vivo*, we are optimistic that additional cases can be identified. The oxidized cysteines observed in crystal structures, although some may be artifacts, may nonetheless reveal functionally important cysteines, as shown for DJ-1, for example.^65^ Studies that combine proteomics and structural biology to characterize the structural impacts of physiologically relevant cysteine oxidations will be critical to bridge this gap.

## Theoretical and Computational Approaches to Cysteine Oxidation Prediction

The analysis of protein structural data we have described above suggests that the propensity of the cysteine thiol to oxidize to sulfenic, sulfinic, and sulfonic acid is tuned by the local structural environment, as well as environmental conditions such as pH and redox potential. Computational methods can in principle be used to predict this susceptibility to oxidation, in a manner similar to the many methods that have been developed to predict the p*K*_a_’s of titratable chemical groups in proteins. Such methods could be useful in identifying potential biologically relevant sites of regulation by cysteine oxidation and to elucidate biophysical principles underlying the trends and examples discussed above. Despite the fundamental theoretical underpinnings of redox chemistry being well understood, computational predictions of these phenomena remain nascent, which we attribute to limited experimental data quantitatively characterizing the susceptibility to cysteine oxidation, and caveats associated with the interpretation of such data;^28,65,104−107^ technical challenges associated with accurately describing redox processes in complex macromolecules; and, perhaps, a relatively low level of awareness of this aspect of protein biochemistry in the computational chemistry/biology community.

The most accurate description of cysteine oxidation can in principle be provided by quantum mechanics, such as density functional theory (DFT),^108−110^ and mixed quantum mechanics/molecular mechanics (QM/MM)^111−115^ methods. Due to the computational expense of quantum mechanical methods on large molecular systems, most applications have focused on model systems and have provided important foundational knowledge about the structures, electron distributions, thermodynamic stability, and other properties of oxidized cysteines. In principle, such calculations are sufficient to determine all of the parameters needed for molecular mechanics descriptions of sulfenic, sulfinic, and sulfonic acids, including force field parameters such as geometries (e.g., propensity for adopting various side chain rotamer states, as shown in Figure S2) and partial changes (as has been done for CSO and OCS in CHARMM36^116^), as well as the standard state reduction potentials in cases in which the values cannot be obtained experimentally, such as reduction of sulfenic acid to thiol.

As an example of such an approach, previous studies have characterized the vertical ionization potential (IP) of cysteine in the gas phase and in solution,^117,118^ making it possible to infer the energetics for the reduction/oxidation reactions and solvation of cysteines using thermodynamic cycles. Additional work will be necessary, however, to generate consensus parameters for the different oxidation states of cysteines that accurately reproduce experimental observations for a range of different systems. One potentially interesting future approach is to perform quantum mechanical calculations on cysteines, in different oxidation states, within model systems that incorporate environmental factors, such as hydrogen bonding, to better characterize their role in differentially modulating the thermodynamics of different oxidation states and the kinetics of transitions among them. Such calculations could be used to modify current parametrizations in molecular dynamics force fields to improve the description of cysteines in different oxidation states.^119^

Purely classical methods, based on molecular mechanics and dynamics, can provide insight into the role of the protein environment in tuning the redox properties of cysteine. Most published work thus far has focused on predicting the p*K*_a_’s of the thiol group in cysteines, which correlates with its reactivity; i.e., a lower p*K*_a_ implies a more reactive (nucleophilic) cysteine. The most common methods for predicting p*K*_a_’s in macromolecules are based on continuum electrostatics^120,121^ or several varieties of constant pH molecular dynamics (CpHMD).^122−125^ We note, however, that cysteine has presented challenges for p*K*_a_ prediction, even more so than other amino acid side chains,^107^ and thus, theoretical p*K*_a_ predictions are unlikely to be accurate enough to reliably identify reactive cysteines. Nonetheless, it is possible to predict cysteine p*K*_a_’s with qualitative accuracy, for example using DelPhiPKA,^126^ which predicts cysteine p*K*_a_’s with a root-mean-square error (RMSE) of 1.7, lower than that predicted by the null model of 2.7, for a set of 18 experimentally characterized cysteines in 12 proteins.^127^ Knowledge-based approaches produced RMSEs higher than those produced by physics-based approaches when benchmarked against the same set of experimental results,^127^ perhaps in part because the sample size of experimentally characterized systems for Cys p*K*_a_’s remains an order of magnitude lower than for more commonly studied titratable residues (His, Asp, and Glu). In general, the connections between shifts in side chain p*K*_a_ and redox reactivity remain underexplored for cysteines in biological systems.^17,128^

More recently, constant redox potential molecular dynamics (CEMD) have been developed to explicitly estimate the susceptibility of Cys and other redox-sensitive groups to oxidation; because such methods must generally also consider pH and protonation, they are perhaps most accurately described as C(pH,E)MD methods.^129^ Reduction potentials have been experimentally measured for a handful of disulfide bonds in proteins, and free energy calculations^130^ have been carried out to determine the ability to reproduce these data. By contrast, we are unaware of any measured reduction potential for oxidation of cysteine to sulfenic, sulfinic, and sulfonic acids in proteins, making it challenging to benchmark C(pH,E)MD methods. More broadly, while the pH used for *in vitro* experiments is frequently carefully controlled using buffers and reported in publications, the redox conditions for experiments are sometimes poorly controlled and inconsistently reported. Most commonly, experiments use large quantities of reducing reagents like dithiothreitol or TCEP, or none at all, just leaving solutions exposed to air. Even more rare is the experimental study of the interplay between pH and redox regulation, although we note a pair of fascinating studies showing how intertwined these parameters can be.^131,132^ It is our opinion that further experimental and computational efforts, with a high degree of synergy, will be needed to further advance our understanding of cysteine oxidation in proteins.

An additional underexplored aspect of cysteine reactivity is the relationship between the propensity for oxidation and the reactivity with electrophilic small molecules. As discussed above, Cys53 in DJ-1 provides an anecdotal example of a reactive cysteine that can both become oxidized and form covalent adducts with dopamine quinone metabolites resulting from oxidation of dopamine. Other endogenous chemical modifications of cysteine have also been described.^59,82,101,133^ In parallel, Cys-targeted covalent inhibitors have attracted a great deal of interest in recent chemical biology and drug discovery efforts.^27,30^ The role of the local protein environment in tuning these various aspects of cysteine reactivity (oxidation, post-translational modification, and reaction with electrophiles), in addition to variables like pH and redox potential, will be a complex but exciting avenue for investigation.

Finally, we note that phosphomimetic mutations, generally to Asp or Glu, have been useful for interrogating the significance of specific sites of post-translational modification in cellular biology. It seems reasonable to postulate that Asp or Glu might also functionally mimic noncatalytic cysteine sulfinic and sulfonic acids, which have a low p*K*_a_ and thus are expected to be negatively charged under physiologically relevant conditions. We know of little empirical support for this supposition, however, and it is not clear whether cysteine sulfenic acid could be mimicked by any standard amino acid.

## Supporting Information Available

Schematic representation of the protocol for identification of cysteine oxidation states in PDB (Figure S1), side chain dihedral distributions for oxidized cysteine structures (Figure S2), histograms of backbone hydrogen bond acceptors and donors within 3.6 Å of cysteine species in structures (Figure S3), subcellular localization of proteins with oxidized cysteines (Figure S4), structural environment of ACAT1 cysteines (Figure S5), keywords for building Figure S4 (Table S1), data for Figure S4 (Table S2), and a link to a database of structures containing cysteines in different oxidation states (PDF)
