# Supplementary material for: Cysteine Oxidation in Proteins: Structure, Biophysics, and Simulation
Source: Biochemistry. 2022 Sep 26;61(20):2165–76. doi: 10.1021/acs.biochem.2c00349 (PMC9583617; doi:10.1021/acs.biochem.2c00349)
Supplement: Supplementary file 1 — bi2c00349_si_001.pdf [file bi2c00349_si_001.pdf]

Supporting information for

Cysteine oxidation in proteins: structure, biophysics, and simulation

Diego Garrido Ruiz[1†], Angelica Sandoval-Perez[1†], Amith Vikram Rangarajan[1†], Emma L. Gunderson[1], Matthew P. Jacobson[1*]

[1]Department of Pharmaceutical Chemistry, University of California, San Francisco, CA, 94158

*Correspondence: Matthew P. Jacobson matt.jacobson@ucsf.edu

†These authors have contributed equally to this work.

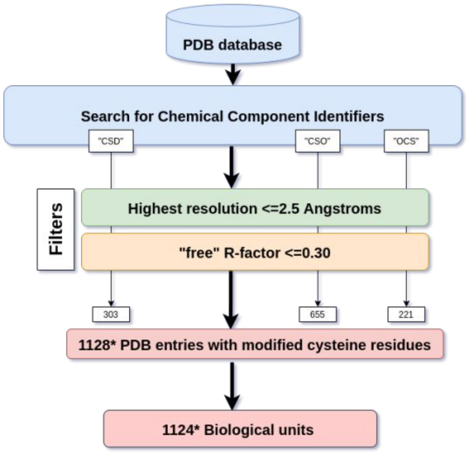

Figure S1. Identification of structures containing oxidized cysteines. Note that some protein structures have multiple oxidized cysteines, such that the final set contains 1128 entries, less than the sum of the three individual search results (1179 entries).

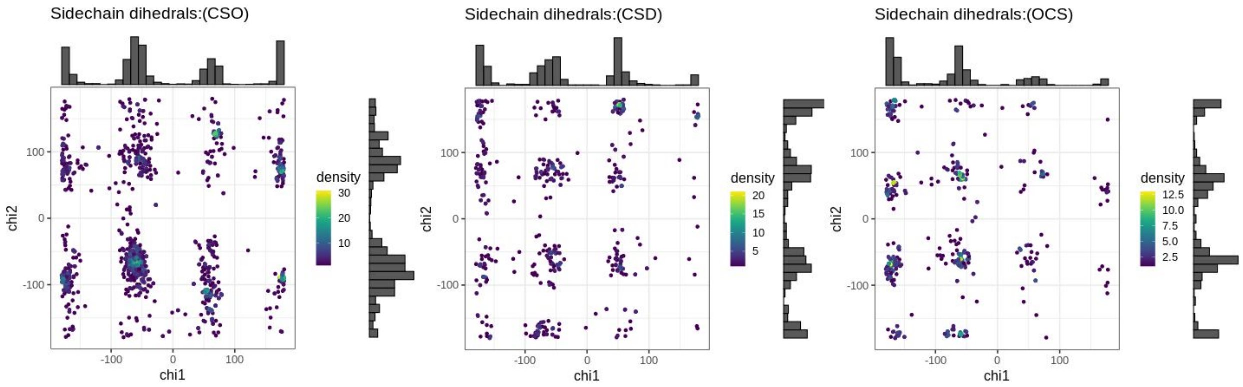

Figure S2. Side chain dihedral angles for modified cysteines.

The dihedral angle chi1 is defined by the C/CA/CB/SG atoms, while chi2 is defined by CA/CB/SG/OD for CSO and CA/CB/SG/OD1 for CSD and OCS.

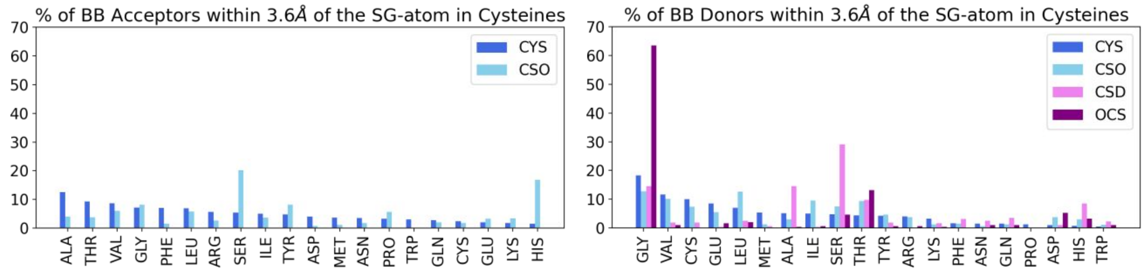

Figure S3. Backbone hydrogen bond acceptors and donors within 3.6 Å of the cysteines' sulfur atom (SG)

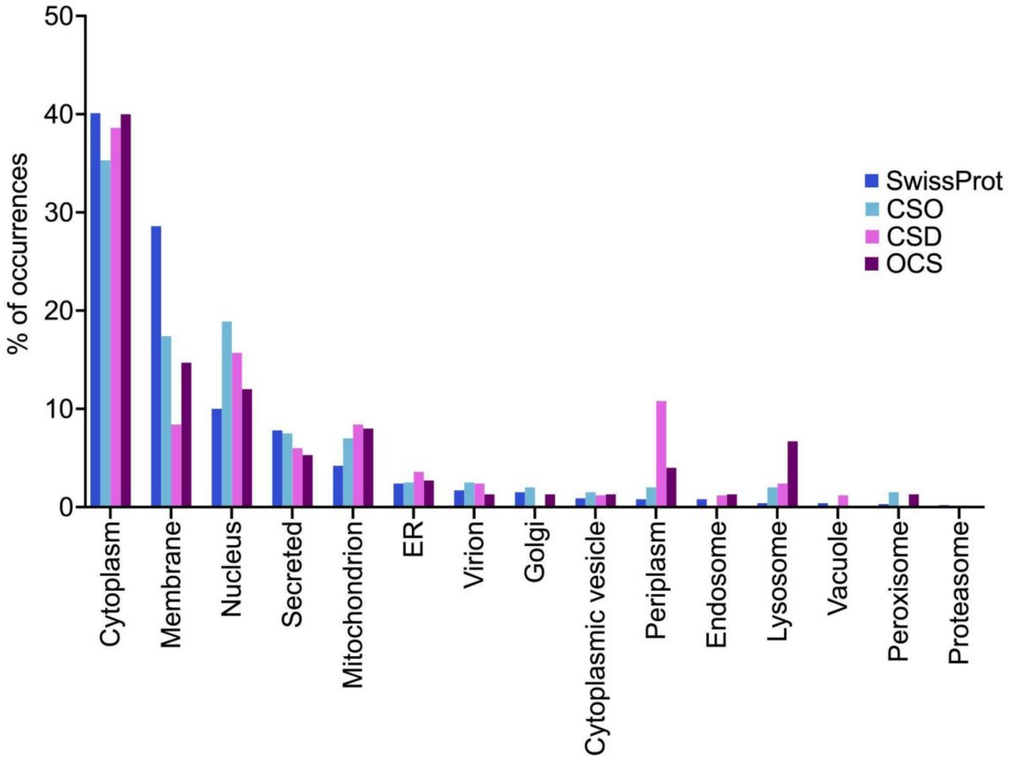

Figure S4. Sub-cellular locations, as provided by UniProt keywords, for proteins in the PDB containing sulfenic (CSO), sulfinic (CSD), and sulfonic (OCS) acids, compared with all SwissProt proteins (manually annotated proteins in UniProt). Some proteins are assigned to multiple locations.

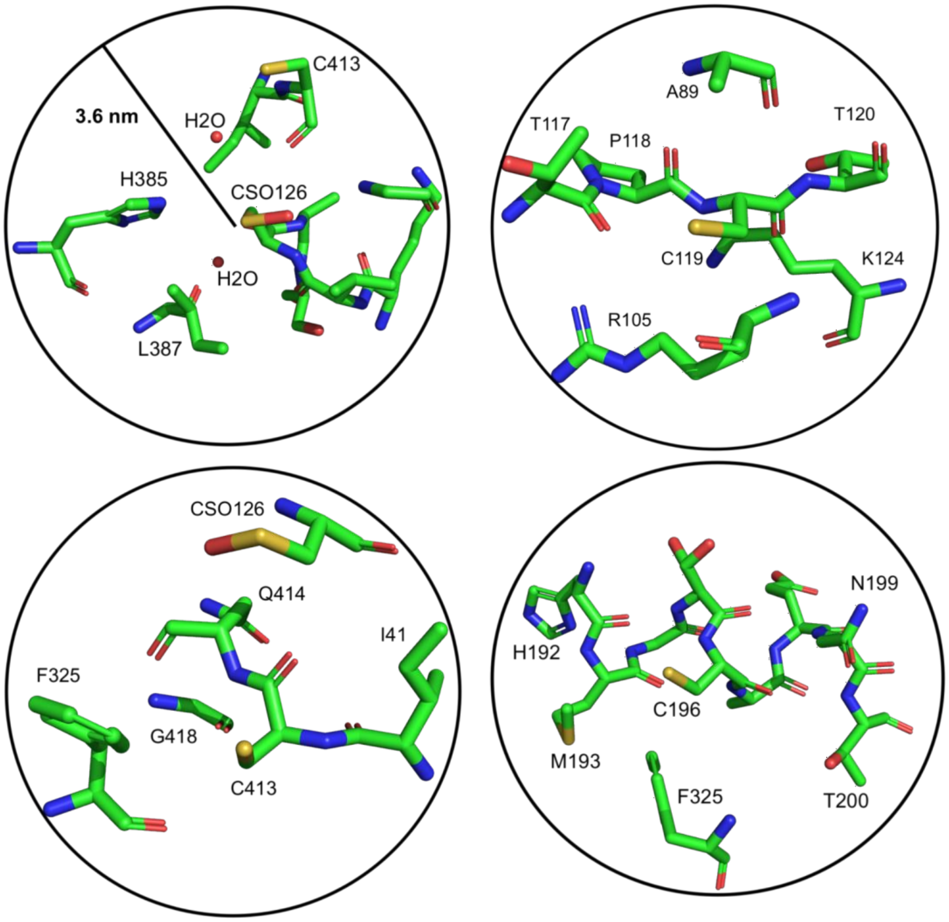

Figure S5. The structural environment of cysteines CSO126, CYS119, CYS413 and CYS196 of the protein Acetyl-CoA acetyltransferase-1 (ACAT1). Amino acid residues and H_2O molecules within 3.6 nm of the respective sulfur atoms are depicted, based on PDB structure 2IBU.

Table S1. UniProt keywords used for building Figure S4.

UniProt Keyword	Cell component
KW-0256	Endoplasmic reticulum
KW-0333	Golgi apparatus
KW-0458	lysosome
KW-0472	Membrane
KW-0496	Mitochondrion
KW-0539	Nucleus
KW-0574	Periplasm
KW-0576	Peroxisome
KW-0647	Proteasome
KW-0926	Vacuole
KW-0946	Virion
KW-0963	Cytoplasm
KW-0964	Secreted
KW-0968	Cytoplasmic vesicle
KW-0967	Endosome

Table S2. Data used to construct Figure S4. Note that some proteins are annotated as observed in multiple compartments, and each annotation is considered.

	CSO(n=142)	CSD(n=57)	OCS(n=48)	Swiss-Prot(n=565255)
ER	5	3	2	9472
Golgi	4	0	1	5909
Lysosome	4	2	5	1680
Membrane	35	7	11	115138
Mitochondrion	14	7	6	16836
Nucleus	38	13	9	40297
Periplasm	4	9	3	3121
Peroxisome	3	0	1	1108
Proteasome	0	0	0	963
Vacuole	0	1	0	1677
Virion	5	2	1	6755
Cytoplasm	71	32	30	161288
Secreted	15	5	4	31391
Cytoplasmic_vesicle	3	1	1	3543
Endosome	0	1	1	3319

Supplementary Data. List of proteins crystallized with cysteines in different oxidation states. PDB structures containing oxidized cysteines from our dataset were mapped to corresponding UniProt identifiers. The SIFTS service[134] was used to identify other PDB structures (with unmodified cysteines) corresponding to each UniProt identifier found in our dataset. This data is presented as a spreadsheet in the github folder. (Jacobson-lab-UCSF/Cysteine_oxidation: Cysteine oxidation in proteins: structure, biophysics, and simulation (github.com)).
